# Assessing the Importance Given by Basketball Coaches to Training Contents

**DOI:** 10.2478/v10078-011-0080-3

**Published:** 2011-12-25

**Authors:** Nuno Leite, Eduarda Coelho, Jaime Sampaio

**Affiliations:** 1 Research Center in Sports Sciences, Health Sciences, and Human Development (CIDESD)

**Keywords:** knowledge, coaching, assessment, experience

## Abstract

The purpose of this study was to compare the importance given by novice, intermediate and experienced basketball coaches to training contents. To achieve this purpose, a sample composed of Portuguese basketball coaches (n = 212) described how they rate the importance of technical, tactical, physical and drill contents. According to the results, there is a wide-ranging differential from novice to experienced coaches. First, while experienced coaches tend to focus on tactical development, novice and intermediate coaches seem to privilege the improvement of technical skills. Second, whereas significant differences between novice and intermediate coaches were found, evidence confirmed that they were higher (both in number and weight) when comparing experienced coaches against novice and intermediate. The study provided strong support to justify the necessity to adjust coaches’ knowledge to players’ biological developmental, and could form the basis of focused interventions in coaching development.

## Introduction

Coaches’ ability to promote effective athletic development and the implications to the quality of sports training has received intense debate among specialists and researchers (Gilbert and Trudel, 2004; Abraham et al., 2006; Martindale et al., 2007). Although coaches’ influence may vary across cultures and sports (Bloom, 1985; Salmela and Moraes, 2003), the presence of a knowledgeable and experienced coach is essential to become an expert performer. Moreover, available literature has been reinforcing the need to adjust coaches’ knowledge and participation not only to the chronologic age but also to the players’ biological development (e.g., stages of athletic development). According to Balyi and Hamilton (1999), experienced coaches should be stimulated to get involved in the initial stages of athletic development. This early involvement may contribute to successfully lengthen the players’ career and be beneficial to the long-term qualitative development of those players (Cushion et al., 2003).

In fact, coaches’ experience is considered the major source of an athlete’s learning (e.g. Gilbert and Trudel, 2001; 2005; Nelson and Cushion, 2006) and could help them promote long-term aims, methods and extensive coherent messages (Bloom, 1985; Gould et al., 2002). Also for these reasons, it is important to plan and stimulate the development of coaches from those who work with children and youth to those who work with elite players. Thus, similarly to what has been suggested for players (Côté et al., 2001; Balyi, 2002), coaches also should pass through several stages of development to attain expertise level. Gilbert et al. (2006) recommend a lifespan perspective focused on developmental paths and activities performed by coaches. However, few studies have quantified the development of sport specific experiences of high-performance coaches, despite suggestions that there are a number of experiential factors that might be consistent in most high-performance coaches’ development (Young et al., 2009). Despite this suggestion, earlier studies on the behaviour of successful coaches (Gilbert et al., 2006) have overlooked the fact that coaches develop not only their specific skills but also their domain-unspecific problem-solving strategies during their long and intensive involvement with the dynamic problems of training and competition. Therefore, coaches’ ability to successfully adapt, modify, and select situations and solve problems is determined not only by a domain-specific knowledge but also by the availability and application of domain-unspecific strategies such as strategic planning (Sternberg, 1998). Moreover, differences in both domain-specific knowledge and domain-unspecific strategies (Abraham et al., 2006), seem to justify problems among coaches in the ability to plan for and implement effective skill development programs (whether it is a long-term planning or a single training session plan).

What is implicit within the concept of coaching expertise is the notion of “added value” that an expert coach can bring to the development of skills (cognitive, motor or emotional). Therefore, the input of a quality coach could provide a structured learning environment that optimizes learning (Abraham and Collins, 1998a; Côté et al., 2003). Hence, it is imperative that the role of the coach in supporting the development of advanced skills is examined to ensure that this added value actually occurs as opposed to a negative value, i.e., the coach who could limit development. While outcome-based coaching competences such as the ability to prepare a training plan, manage a group or demonstrate a skill are important, other competences such as problem solving, decision making, and innovation have been neglected in coaches education. The focus has been on what and how coaches do (procedural knowledge) as opposed to why they do it (declarative knowledge).

Recently, Abraham et al. (2006) suggested the implementation of a coaching model, coaching schematic, which could reflect the coaching process from both a content and information-processing stance. Throughout this model, it becomes obvious that it is declarative knowledge, or understanding of a situation that gives broad procedural rules their flexibility and transferability and allows appropriate actions/solutions (i.e., specific procedural knowledge) to be implemented in a given situation (Abraham and Collins 1998b; Pennington et al., 1995). Considering that declarative knowledge is actually developed through explicit cognitive elaborations of experience (Pennington et al., 1995), this could mean that these experiences could result in a highly effective knowledge base to be used both in planning and monitoring players’ development. Therefore, declarative knowledge contributes to differentiate coaches’ ability to be consistently innovative, introducing new goals or using the same drill with different purposes.

Specifically in team sports, effective players must have a comprehensive understanding of their own performance and its effect on teammates or opponents (McPherson 1994; Williams and Davids, 1995). Thus, coaches must consider the variety and complexity of skills required and the unpredictability of conditions under which all of these skills and decisions are produced and made (Abraham et al., 2006). This should lead the coach to question and consider how much variety and unpredictability is required in the training sessions that are planned. Furthermore, since learning does not happen immediately, this consideration should take account of the learning environments required over long periods of time and several training sessions. However, while most studies have focused on coaches behaviour during training sessions and during competitions (Smith and Cushion, 2006), there is still a lack of peer reviewed studies examining the importance given by coaches to training contents. While the available literature suggests severe differences from novice to expert coaches across the spectrum of team sports (Balyi and Hamilton, 2004), coach qualification level, and player age and abilities, there is a limited comprehensible explanation on coaches’ perceptions about the importance of training factors and drills used in players’ development. In view of the facts exposed above it was hypothesized that there is a wide-ranging differential from novice to experienced Portuguese basketball coaches and that differential could improve the understanding of the developmental process that leads to coaching expertise in basketball.

## Material and Methods

### Participants

Two hundred and twelve Portuguese basketball coaches volunteered to participate in the study. The sample was divided according to coaches’ level (obtained via basketball coaching certificates, organized by the Portuguese Basketball Federation): level 1, novice (n = 88, age M = 22.6, S.D. = 4.0, years of experience M = 4.8, S.D. = 2.2), level 2, intermediate (n = 75, age M = 29.2, S.D. = 6.1, years of experience M = 8.3, S.D. = 6.3) and level 3, experienced (n = 49, age M = 41.5, S.D. = 7.6, years of experience M = 19.8, S.D. = 9.9). Coaches were also grouped across developmental stages suggested by Balyi and Hamilton (2004) in which their players were involved at the time of this study: Fundamentals (6 to 10 years, n = 54), Learning and Training to Train (10–14 years, n = 52), Training to Compete (14 –18 years, n = 68) and Training to Win (18 years and beyond, n = 38). However, the distribution of the participants across developmental stages restricted inferential analysis due to the limited number of level 3 coaches working with players between 6 and 10 years of age (n = 2), and level 1 coaches working with players aged 18 and beyond (n = 1). Despite this limitation, one interesting finding emerged from this distribution. Essentially, results suggested the existence of an inverse relationship between the developmental stage and the coaching education level. Accordingly, the majority of coaches working with players between 6 and 10 years of age had level 1 (n = 44). On the other hand, only 2 (Fundamentals), 4 (Learning and Training to Train) and 11 (Training to Compete) coaches had the higher level (e.g., level 3). Ultimately, the less experienced coaches seem to be the ones assuming the responsibility of the initial stage of development.

### Methods

Although it is acknowledged that there are numerous ways of evaluating knowledge (Saury and Durand, 1998), this study views knowledge as a lifespan construction, a part of coaches’ education and development, trying to better understand how such knowledge could evolve and influence coaches perceptions and behaviours. In this study, the importance given to training contents was assessed through a questionnaire where the participants described how they rate the importance of 23 items grouped in four-domains, specifically, (i) four technical-related items: basic movements (such as offensive ready stance, running, changing direction/speed, stopping, pivoting and jumping, specific movements (triple threat position, pivot, face up or one and two phase stops), and technique fundamentals (dribbling, passing and shooting), and basic defensive movements (defensive stance, defensive slide, denial defence and box-out); (ii) six tactical-related items: small sided games (1×1, 2×2, 3×3, 4×4), offensive superiority games (2×1, 3×2, 4×3), defensive superiority games (1×2, 2×3, 3×4), match (5×5), offense and defense (understood as game phases); (iii) two physical-related items: conditioning (strength, endurance and flexibility) and coordinative (agility, balance, coordination and speed), (iv) eleven drill-related items: opposition, competition, repetition, execution speed, execution technique, length, timing, decision-making, space, game and enjoyment. The questionnaire contained detailed information regarding each domain and examples for each item-domain listed, (similar to the example presented regarding the technical-related items).

The questionnaire was validated by sports training experts through four different steps: (i) first, a preliminary version of the questionnaire was designed, supported by available scientific research; (ii) the second step was the verification of the importance, clarity and understanding of those questions, and simultaneously, to the quantity and intervals of the answers. To accomplish this purpose, five specialists in coaching intervention and education and five specialists in training methodology were asked to examine that preliminary version of the questionnaire; (iii) after the examination of those specialists some of the initial questions were excluded and only those questions that had the agreement of at least four of the five specialists were considered to the final version; (iv) the final version of the questionnaire was designed and applied to the coaches.

A confirmatory factor analysis was performed. The tested model was a four-dimension correlated structure. The method of estimation maximum likelihood was used because it is robust to violation of multivariate normality (Chou and Bentler, 1995).The results showed that 20 of the path coefficients of latent variables were significant at 0.001 level. Only defensive superiority games and game items were significant at level 0.05. The enjoyment item obtained a standardized regression weight (factor loading) of 0.116, with an insignificant value (p = 0.121). Despite this fact, the authors decided to maintain this item due to its relevance in the theoretical construct of the study. Yet, the results obtained in this item must be interpreted with caution. All the correlations between the four factors were significant, with values ranging between 0.265 and 0.704. Regarding the homogeneity of the items within the four factors, this questionnaire demonstrated a reasonable internal consistency, with Cronbach alpha coefficients ranging between 0.69 and 0.75.

### Procedures

Questionnaire completion was conducted individually in a quiet environment, lasting approximately 30 minutes. The answers were chosen by the coach from a set of alternatives supplied by the authors using a 5-point Likert scale (1 = rarely present in drills used in training sessions: 0–20% of the drills, 2 = unusually present in drills used in training sessions: 21 to 40%, 3 = present in drills used in training sessions: 41–60%, 4 = frequently present in drills used in training sessions: 61–80%, 5 = always present in drills used in training sessions). Note that these perceptions should typify coaches’ current beliefs. Reliability of the answers provided by the coaches was tested through temporal stability of the measures, thus, 10% of the sample (randomly selected) was asked to refill the questionnaire one month after the first data collection (n = 25). To examine the correspondence between the answers given by the coaches at both time points the percent agreement (Bahrick et al., 1996) was computed. There was a high level of agreement (97%) between the information given by the coaches in both moments. These results indicate that data was reliable.

### Statistical Analysis

A discriminant analysis was performed in order to determine: i) which of the obtained variables are more useful in predicting coaches’ ability; ii) the mathematical equation that enhanced differences in variable means between novice, intermediate and experienced coaches, and, iii) the accuracy of the equations. Assumptions on discriminant analysis were for independency amongst variables, multivariate normal distribution and equal variance-covariance across groups (Silva and Stam, 1995). The interpretation of the obtained discriminant functions was based on examination of the structure coefficients greater than 0.30, meaning that variables with higher absolute values have a powerful contribution to discriminate between groups (Pedhazur, 1982; Mueller and Cozad 1993).

Validation of discriminant models was conducted using the leave-one-out method of cross-validation (Norušis, 1993). Cross-validation analysis takes subsets of data for training and testing and is needed in order to understand the usefulness of discriminant functions when classifying new data. This method involves generating the discriminant function on all but one of the participants (n - 1) and then testing for group membership on that participant. The process is repeated for each participant (n times) and the percentage of correct classifications generated through averaging for the n trials. The statistical analyses were performed using SPSS software release 16.0 (SPSS Inc., Chicago, IL) and significance was set at p ≤ 0.05.

## Results

The means, standard deviations, and structure coefficients for each group of basketball coaches for the studied domain-related items are displayed in Table 1. Globally, the higher mean values corresponded to the technical items and the lower values to the physical items. Both novice and intermediate coaches’ higher values were found at technical items, while the experienced gave more importance to tactical items (p< 0.01). Individually, the higher values were obtained in the technical fundamentals for all levels. On the opposite hand, it was found that the lowest values were attributed to defensive superiority games also in the three groups. Concerning the physical items, results showed that novices privileged agility and coordination) while experienced coaches gave higher importance to conditioning (strength, endurance, etc.). The results for drill items demonstrated that game (M = 4.33, S.D = 0.69) was the most valuated, whereas the lowest corresponded to enjoyment (M = 3.42, S.D = 1.22). Both novice and intermediate coaches endorsed higher importance to game, while experienced coaches gave more importance to competition.

Novices’ lowest value was attributed to opposition, intermediates lowest value was attributed to length and experienced lowest value was attributed to enjoyment.

**Figure 1:**
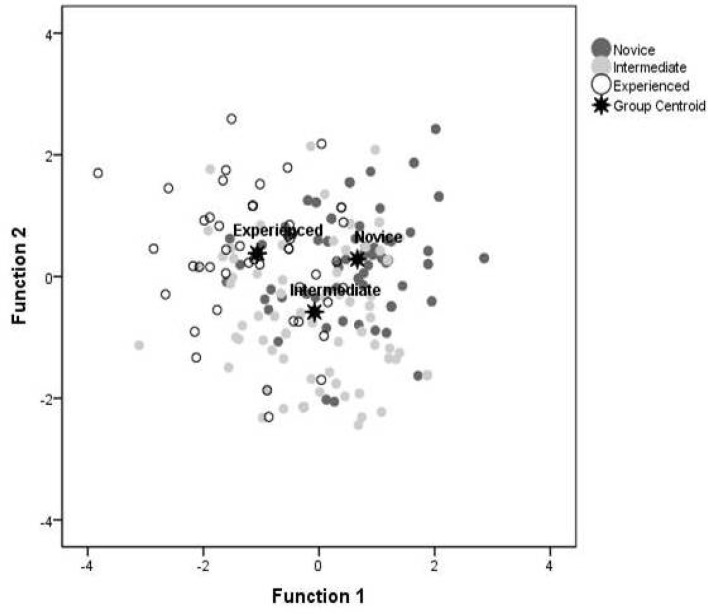
Territorial map of the coaches relative to their skill position representing how widely dispersed the centroids are from one another in standardized discriminant scores. The points indicate the group centroid for novice, intermediate and experienced coaches.

As shown in Figure 1, the group centroid distances (especially for the first discriminant function) and structure coefficients, describe the domain-related item profiles that differentiate between novice, intermediate and experienced coaches studied. The structure coefficients quantify the potential of each domain-related item to maximize differences between means amongst novice, intermediate, and experienced coaches. Accordingly, the larger the magnitude of the coefficient, the greater the contribution of that item to the discriminant function. Discriminant function 1 accounted for 70.5% of the variance, whilst discriminant function 2 accounted for the remaining 29.5%. Results from function 1 reflect an emphasis on enjoyment and a de-emphasis on offense, defense, opposition and competition. On the other hand, results from function 2 reflect an emphasis on decision-making and length and a de-emphasis on basic defensive movements, offensive and defensive superiority games (Table 1).

The leave-one-out test summarizes the ability of the discriminant functions to correctly classify the players in their respective positions. This analysis provided an overall percentage of successful classification of 62.3% of all coaches, with 73.9% for novice, 52.0% for intermediate and 57.1% for experienced were correctly classified on the basis of their domain-related items.

## Discussion

The purpose of this study was to compare the importance given by novice, intermediate and experienced Portuguese basketball coaches to training contents. The results confirmed the importance of technical fundamentals, especially those performed with the ball (e.g. passing, dribbling, and shooting), regardless of the coaches’ level or experience. Additionally, novice and intermediate coaches tend to focus and reinforce the importance of appropriate development of technical items. On the other hand, experienced coaches declared tactical items as more important in the players’ development. These results confirmed different wide-ranging perceptions of the long-term development of basketball players. Considering these facts, especially the higher importance given by more experienced coaches to tactical items, this study could confirm some of the assumptions of the Teaching Games for Understanding model (Bunker and Thorpe, 1982; Kirk and MacPhail, 2002). In fact, while the traditional approaches to teaching/learning in team sports, such as basketball, are primarily focused in the development of technique (Rink, 2001), recent expansion of tactical-dominant models have contributed to redefine team sports teaching/learning. In this particular approach, players are stimulated to develop tactical awareness and therefore skill execution is always in a direct relation with the players’ performance in game-like situations. Consequently, the foundations of this model suggest that in early stages players should be confronted with tactical problems, helping them to develop their comprehension of the game and leading them to understand the need to optimize their skills (Mitchell, 1996; Cushion, 2002) in a game environment (Turner and Martinek, 1995). For these reasons, the results of this study enhance the importance of tactical development, and perhaps more importantly, reinforces the need to rethink the models used by less skilled or inexperienced coaches when working with young players. It may be arguable that experienced coaches that contributed to this study work preferentially at the high sports level (e.g., professional leagues and national teams) and therefore have a perception based on what it takes to win at that level. However, this does not invalidate that those subjects are also the most experienced coaches and have a better understanding of what is more important to consistently develop a basketball player. Another explanation that could help understand significant differences between novice and experienced coaches may be that experienced coaches evaluate these skills as being already developed and therefore their concerns could be more focused on technical optimization. Results obtained in drills, specifically execution speed and technique may also be supportive of these arguments, since experienced coaches rated the speed of execution higher while novice concerns are more focused on technique.

Results also confirmed the defensive superiority games as the less important in the players’ development in the three coaches groups. Despite the crucial role of small-sided games in the coaching process, confirmed by the results of this study and well documented in recent scientific literature (Hill-Haas et al., 2008), available studies are limited when related to the importance of superiority or inferiority games. More importantly, literature is scarce when we try to establish a proper rationale between these items and the needs of basketball players’ development. Usually, defensive superiority games, such as 1×2 or 2×3, are complex game-like situations which are related with the development of team defensive strategies and therefore, more specific to higher levels of competition. For these reasons, it is not difficult to understand the lower results obtained in this item, especially those corresponding to novice and intermediate coaches. The same reason could help understand differences found in both offensive and defensive team work. Thus, while experienced coaches recognize the importance of tactical development to beat the opponents and be successful, novice coaches are more concerned with technical development.

Results also showed diverse approaches in physical items. While novice coaches, probably influenced by the fact that they are usually involved in the initial stages of athletic development, recognized higher importance to capacities of which optimal windows of trainability are placed in earlier ages (Balyi, 2002) experienced coaches gave higher rates to conditioning. Nowadays, it is consensual that the development of predominantly conditioning or coordinative abilities does not induce the same effect or adaptation in players at different ages (Stafford, 2005). Thus, those who were involved with children or youth (consequently more susceptible to develop coordinative abilities) seem to prefer the development of those type of physical items (Malina et al., 2005). Conversely, it is reasonable that experienced coaches, usually linked with elite players, dedicate more time to the appropriate development of strength, speed or endurance (Blimkie and Bar-Or, 1996). Finally, several studies (Helsen et al., 2000; Malina et al., 2000; Glamser and Vincent, 2004) suggest that besides adjusting the stimulus induced to the maturational development of the players, physical loads should be integrated with technical and tactical training (Memmert and Roth, 2007).

The increasing competitive demands from youth sport to high-level performance may help explain the results obtained in several drill items like opposition, competition, execution speed, timing, decision-making and game. In fact, while in the initial stages of the players’ development (where inexperienced coaches are more often involved) these items are valuable but are not priorities, success in high-level competitions highly depends on the ability to beat the opponents, making better and quicker decisions throughout the game. Moreover, as the final score is almost the only measure of success at this level, it is understandable that experienced coaches highly rank those drill items. Thus, these results should benefit the debate among team sports coaches, in order to increase the quality of the sports training and promote an effective athletic development related with expert performers’ models. Selecting drills where game-like situations are more frequent (Strean and Holt, 2000), where cooperation and opposition occur in a dynamic interaction, stimulating the ability to execute skills within the right moment (Gréhaigne et al., 2001) and encouraging tactical awareness, expressed by the constant need to make proper decisions (McPherson and Kernodle, 2003), can benefit the development of the tools needed to achieve a higher level of performance. However, teaching players to make good and quick decisions is not an easy task (Turner and Martinek, 1995). What this study confirms is that, according to the importance given by experienced coaches, it is crucial to anticipate that stimulus, benefiting the development of young players (Thomas et al., 2001). Considering Elferink-Gemser et al. (2007) suggestions, future experts exhibit distinguishing attributes in tactical skills such as decision-making around 14 years of age, however, we may find support to justify and encourage an earlier development of these skills.

Finally, the results also supported coaches’ different approaches regarding the importance given to enjoyment within practice drills. Available research suggests that contrary to what happens with expert performers, youngsters start practicing sport mainly because of intrinsic motivations (Wall and Côté, 2007). Developmental models such as the Development Model for Participation (Côté, 1999; Côté and Hay, 2002) or Long-Term Athlete Development (Balyi and Hamilton, 2004) suggest that during the initial stages, coaches should maximize participation, fun and enjoyment. On the other hand, advanced stages of athletic development should focus on performance development. The results of this study seem to confirm these statements, as novice and intermediate coaches gave high importance to enjoyment within practice drills.

## Figures and Tables

**Table 1:** Descriptive results for each domain-related item (values are M±S.D.), discriminant function structure coefficients and tests of statistical significance.

					**Discriminant Analysis**	
	**Items**	**Novice**	**Intermediate**	**Experienced**	**Function 1**	**Function 2**
**Technical** 4.14±0.81	Basic movements	3.99±0.88	3.92±0.87	3.57±1.21	0.24	−0.13
	Specific movements	4.28±0.73	4.28±0.78	4.06±1.05	0.15	−0.12
	Technique fundamentals	4.35±0.79	4.45±0.64	4.61±0.75	−0.20	0.02
	Basic defensive movements	3.78±1.09	4.24±0.84	4.06±0.94	−0.19	−0.38

**Tactical** 3.93±0.92	Small sided games	4.17±0.83	4.17±0.80	4.41±0.64	−0.17	0.14
	Offensive superiority games	4.02±0.90	4.27±0.76	3.90±0.96	0.06	−0.38
	Defensive superiority games	3.03±1.03	3.37±1.01	2.98±1.03	0.01	−0.39
	Match (5×5)	3.95±1.05	3.96±0.81	4.08±1.04	−0.07	0.06
	Offense	3.89±1.01	4.09±0.77	4.41±0.67	−0.35	0.02
	Defense	3.84±1.15	4.09±0.83	4.41±0.64	−0.35	−0.01

**Physical** 3.76±1.08	Conditioning	3.52±1.20	3.92±0.85	3.94±0.97	−0.25	−0.23
	Coordination	3.84±1.22	3.72±0.94	3.63±1.19	0.11	0.03

**Drills** 3.96±0.85	Opposition	3.73±0.91	3.89±0.76	4.18±0.78	−0.32	0.04
	Competition	3.89±0.78	4.08±0.83	4.45±0.68	−0.42	0.07
	Repetition	3.99±0.94	4.01±0.78	4.06±0.90	−0.05	0.01
	Execution speed	3.99±0.95	3.99±0.83	4.24±0.80	−0.16	0.14
	Execution technique	4.25±0.97	4.29±0.77	4.16±0.80	0.05	−0.10
	Length	3.82±0.74	3.53±0.78	3.73±0.73	0.09	0.37
	Timing	3.76±0.84	3.72±0.95	4.14±0.71	−0.24	0.26
	Decision-making	3.84±0.77	3.69±0.85	4.27±0.79	−0.28	0.45
	Space	4.16±0.79	4.11±0.80	4.20±0.71	−0.03	0.10
	Game	4.32±0.72	4.33±0.66	4.37±0.67	−0.04	0.01
	Enjoyment	3.98±1.09	3.24±1.18	2.69±1.03	0.68	0.19

			Wilks’ Lambda		0.58	0.84
			Chi-Square		108.5	34.5
			*P*		<0.001	<0.05
			Eigenvalue		0.45	0.19
			Relative percentage		70.5	29.5
			Canonical correlation		0.56	0.40
